# COVID Vaccine Rollout for Older Adults in Israel

**DOI:** 10.1093/geroni/igab046.714

**Published:** 2021-12-17

**Authors:** Naim Mahroum

**Affiliations:** Sabar Health, Even Yehuda, Tel Aviv, Israel

## Abstract

The COVID vaccine rollout in Israel has prioritized older adults. It led to a substantial decline in the incidence of COVID-19 in older adults. The new variants are threats to the current achievements. We will provide detailed information later. The symposium has experts from 8 countries. We will use an interview and dialog format, instead of presentations.

